# Supporting clinical trials through healthcare informatics

**DOI:** 10.1186/1745-6215-16-S2-O67

**Published:** 2015-11-16

**Authors:** Claire Jones, Emily Jefferson, Fiona Hogarth, Roberta Littleford, Margaret Band

**Affiliations:** 1 University of Dundee, Dundee, UK

## 

Administration of trials involving large numbers of participants, coordinating multi-centred sites or drug management can all result in major logistical challenges for any trial. The enormity of these challenges can be drastically reduced through the adoption of healthcare informatics.

ECLS (Early Cancer detection test - Lung cancer Scotland) is a high impact study with the aim of detecting often-deadly lung cancer at an earlier and more treatable stage. This project, a collaboration between researchers at the Universities of Dundee, Glasgow and Nottingham, NHS Scotland, the Tayside Clinical Trials Unit (TCTU) and supported by the Health Informatics Services Centre (HIC), provides a prime example of how healthcare informatics can be successfully applied through the full life cycle of a trial:

• A web based Patient Recruitment system is being utilised to invite ~50,000 potential participants to take part in this study. This provides the facility to track participant progress, generate online reports and record participant appointments.

• A Letter App supports the generation of letters as required by the study (e.g. appointment/result letters).

• The Tayside Randomisation System (TRuST) balances the randomisation allocation for this study based on a minimisation with stratification algorithm. TRuST can also be utilised for allocation clustering preventing potential contamination and drug management offering full accountability.

• On completion of recruitment the study will employ the services of the HIC Data Linkage Service to follow up participants and link to their routinely collected electronic health records.

• HIC Services further supports clinical trials through the creation of study websites and data collection tools.

